# Differences in Discounting Behavior and Brain Responses for Food and Money Reward

**DOI:** 10.1523/ENEURO.0153-23.2024

**Published:** 2024-04-03

**Authors:** M. Markman, E. Saruco, S. Al-Bas, B. A. Wang, J. Rose, K. Ohla, S. Xue Li Lim, D. Schicker, J. Freiherr, M. Weygandt, Q. Rramani, B. Weber, J. Schultz, B. Pleger

**Affiliations:** ^1^Department of Neurology, BG University Clinic Bergmannsheil, Ruhr-University Bochum, Bochum 44869, Germany; ^2^Institute of Cognitive Neuroscience, Faculty of Psychology, Ruhr-University Bochum, Bochum 44801, Germany; ^3^Firmenich SA, Satigny 1242, Switzerland; ^4^NutriAct-Competence Cluster Nutrition Research Berlin-Potsdam, Nuthetal 14558, Germany; ^5^Cognitive Neuroscience (INM-3), Institute of Neuroscience and Medicine, Research Center Jülich, Jülich 52428, Germany; ^6^Sensory Analytics & Technologies, Fraunhofer Institute for Process Engineering and Packaging IVV, Freising 85354, Germany; ^7^Department of Psychiatry and Psychotherapy, Friedrich-Alexander-Universität Erlangen-Nürnberg, Erlangen 91054, Germany; ^8^Experimental and Clinical Research Center, a cooperation between the Max Delbrück Center for Molecular Medicine in the Helmholtz Association and Charité Universitätsmedizin Berlin, Berlin 10115, Germany; ^9^Charité—Universitätsmedizin Berlin, Corporate Member of Freie Universität Berlin and Humboldt-Universität zu Berlin, Experimental and Clinical Research Center, Berlin 13125, Germany; ^10^Max Delbrück Center for Molecular Medicine in the Helmholtz Association (MDC), Berlin 13125, Germany; ^11^Center for Economics and Neuroscience (CENs), University of Bonn, Bonn 53113, Germany; ^12^Institute of Experimental Epileptology and Cognition Research (IEECR), University of Bonn, Bonn 53127, Germany

**Keywords:** attribute-wise models, computational modeling, delay discounting, neuroimaging, option-based models, primary and secondary reward

## Abstract

Most neuroeconomic research seeks to understand how value influences decision-making. The influence of reward type is less well understood. We used functional magnetic resonance imaging (fMRI) to investigate delay discounting of primary (i.e., food) and secondary rewards (i.e., money) in 28 healthy, normal-weighted participants (mean age = 26.77; 18 females). To decipher differences in discounting behavior between reward types, we compared how well-different option-based statistical models (exponential, hyperbolic discounting) and attribute-wise heuristic choice models (intertemporal choice heuristic, dual reasoning and implicit framework theory, trade-off model) captured the reward-specific discounting behavior. Contrary to our hypothesis of different strategies for different rewards, we observed comparable discounting behavior for money and food (i.e., exponential discounting). Higher *k* values for food discounting suggest that individuals decide more impulsive if confronted with food. The fMRI revealed that money discounting was associated with enhanced activity in the right dorsolateral prefrontal cortex, involved in executive control; the right dorsal striatum, associated with reward processing; and the left hippocampus, involved in memory encoding/retrieval. Food discounting, instead, was associated with higher activity in the left temporoparietal junction suggesting social reinforcement of food decisions. Although our findings do not confirm our hypothesis of different discounting strategies for different reward types, they are in line with the notion that reward types have a significant influence on impulsivity with primary rewards leading to more impulsive choices.

## Significance Statement

The objective of the research was to explore the impact of the type of reward on decision-making processes. By utilizing delay discounting in conjunction with the hierarchical Bayesian modeling, the study unveiled that participants employed similar discounting strategies but exhibited varying levels of impulsivity based on the type of reward they received. Specifically, it was observed that monetary rewards were discounted at a less steep rate compared with food rewards, particularly when considering longer time delays. This difference can possibly be attributed to the fact that food is perishable, whereas money retains its long-term value stability.

## Introduction

Why some can refrain from tempting rewards and others cannot remains a puzzling question. The ability to control emotions and desires and to suppress behaviors not in line with our goals is termed self-control (Baumeister et al., 2007; Flanigan and Climie, 2020). The inability to refrain from tempting rewards associated with a lack of consideration for potential negative consequences relates to impulsivity (Duckworth and Kern, 2011; Flanigan and Climie, 2020). Therefore, individuals with weak self-control are more driven by incentive impulses than those with strong self-control (Friese and Hofmann, 2009). Unsurprisingly, high levels of impulsivity are also linked to addictive behaviors (Lee et al., 2018). More specifically, it has been found that impulsive individuals are more inclined to internet (Cao et al., 2007; Choi et al., 2014), social media (Cudo et al., 2020), smartphone (Jo et al., 2018; Zhu et al., 2021), gaming, and gambling addictions (Castellani and Rugle, 1995), as well as larger calorie intake (Guerrieri et al., 2007a,b) and higher eating frequency (Clarke, 2012).

To what extent such disadvantageous or even dangerous reward behavior translates to other types of reward remains a matter of debate. In the current study, we hypothesized that individuals who decide either on primary (i.e., food) or secondary reward (i.e., money) employ different types of choice behavior, processed in (partly) different brain networks.

A common way to assess reward-based choice behavior in healthy individuals and psychiatric diseases (Amlung et al., 2019; Bickel et al., 2019; Lempert et al., 2019; Odum, 2020) is delay discounting (DD; or temporal discounting), specifically designed to assess the relative valuation of receiving a reward at an earlier timepoint compared with receiving a higher reward at a later timepoint (Frederick et al., 2002; Sosa and dos Santos, 2018). Given two rewards of similar magnitude, one earlier and one delayed, the earlier reward is generally preferred (Chung and Herrnstein, 1967); however, with increasing reward amount and decreasing delay, the delayed option becomes increasingly attractive, up to an indifference point where the smaller earlier reward is chosen equally often as the larger delayed one. Mapping a hyperbolic function through these points using one free variable *k* represents a widely accepted measure of impulsivity, with high cross-species, cross-population, and intraindividual consistency (Odum, 2011, 2020). Beyond the indifference point, the delayed reward is increasingly preferred, due to reduced demands of self-control over the general tendency to react impulsively.

In this study, we used functional magnetic resonance imaging (fMRI) to investigate DD of primary (i.e., food) and secondary reward (i.e., money) in young and physically and mentally healthy non-obese and non-addicted human participants. To decipher differences in behavioral strategies for food and money reward, we applied a variety of discounting models to capture differences in behavioral strategies, such as classical exponential and hyperbolic discounting models, as well as the three recently proposed attribute-wise choice models (heuristic models; Read et al., 2013; Scholten et al., 2014; Marzilli Ericson et al., 2015).

We hypothesized that option-based models capture the rather simple “now-or-later” food and money decisions of our task better than the more complex attribute-wise models. For primary reward (i.e., food), we expected a stable decrease of relative value over time—best captured by exponential discounting—whereas for secondary reward (i.e., money), we assumed that the reward value for long delays decreases less strongly as for food, best captured by hyperbolic discounting. To assess reward-type-related brain responses, we used fMRI and compared the brain activity elicited by food and money discounting. We expected that differences between food and money discounting relate to altered activity in the dorsolateral prefrontal cortex (dlPFC), involved in executive control (Funahashi, 2001; Wagner et al., 2001); the ventromedial prefrontal cortex (vmPFC), as a value-coding region (Levy and Glimcher, 2012; Bartra et al., 2013); and the ACC, which was shown to be associated with choice difficulty (Botvinick et al., 2001; Botvinick, 2007; Pochon et al., 2008; Shenhav et al., 2014; Vassena et al., 2017).

## Materials and Methods

### Participants

Thirty-seven lean and healthy participants were recruited (body mass index, BMI (weight (kg) / height (m)^2^; mean 24.43 ± 6.05 standard deviation; range, 18–25 kg/m^2^)). After we excluded food or game addiction, with the German versions (Cremer et al., 2001; Meule et al., 2012) of the Yale Food Addiction Scale (Gearhardt et al., 2009) and South Oaks Gambling Screen questionnaires (Lesieur and Blume, 1993), contraindications to MRI scanning were checked (i.e., metal implants, claustrophobia, pregnancy, breastfeeding, large tattoos). Present or past neuropsychiatric and other chronic diseases, regular drug intake, and vegetarians/vegans were further exclusion criteria. Eligible participants signed an informed consent approved by the ethics committee at the Ruhr University Bochum (application 548). All participants were asked to refrain from eating 4 h before the experiment. They additionally rated their hunger level on a visual analog scale by making a cross on a 10 cm long line with the end points “not hungry at all” and “extremely hungry.” The distance between the end point “not hungry at all” and the cross was 6.5 ± 0.2 cm (mean ± standard error), indicating that participants have been relatively hungry while doing the task.

Seven participants had to be excluded because they have shown no systematic discounting behavior, choosing either the immediate or delayed reward in over 90% of the trials. Two other participants were excluded because of extremely long reaction times (>12 s) on most of the trials suggesting loss of focus. The data of the remaining 28 participants (mean age = 26.77 ± 9.56 years; mean BMI = 22.75 ± 2.03; 18 females) was analyzed. Excluding participants did not affect the results of model comparison. We, however, found less significant results for the entire sample, suggesting that excluded participants increased noise.

### Barratt Impulsiveness Scale (BIS-15)

Participants’ self-reported level of impulsivity was assessed with the German short version (Meule et al., 2011) of the BIS-15 (Spinella, 2007). Participants were asked to rate 15 items from 1 (rarely/never) to 4 (almost/always), belonging to three subscales: attentional, motor, and non-planning impulsivity. General impulsivity, of particular interest for this study, was indexed by the total score summarizing the 15 items of the questionnaire.

### DD fMRI task

**Figure 1.**
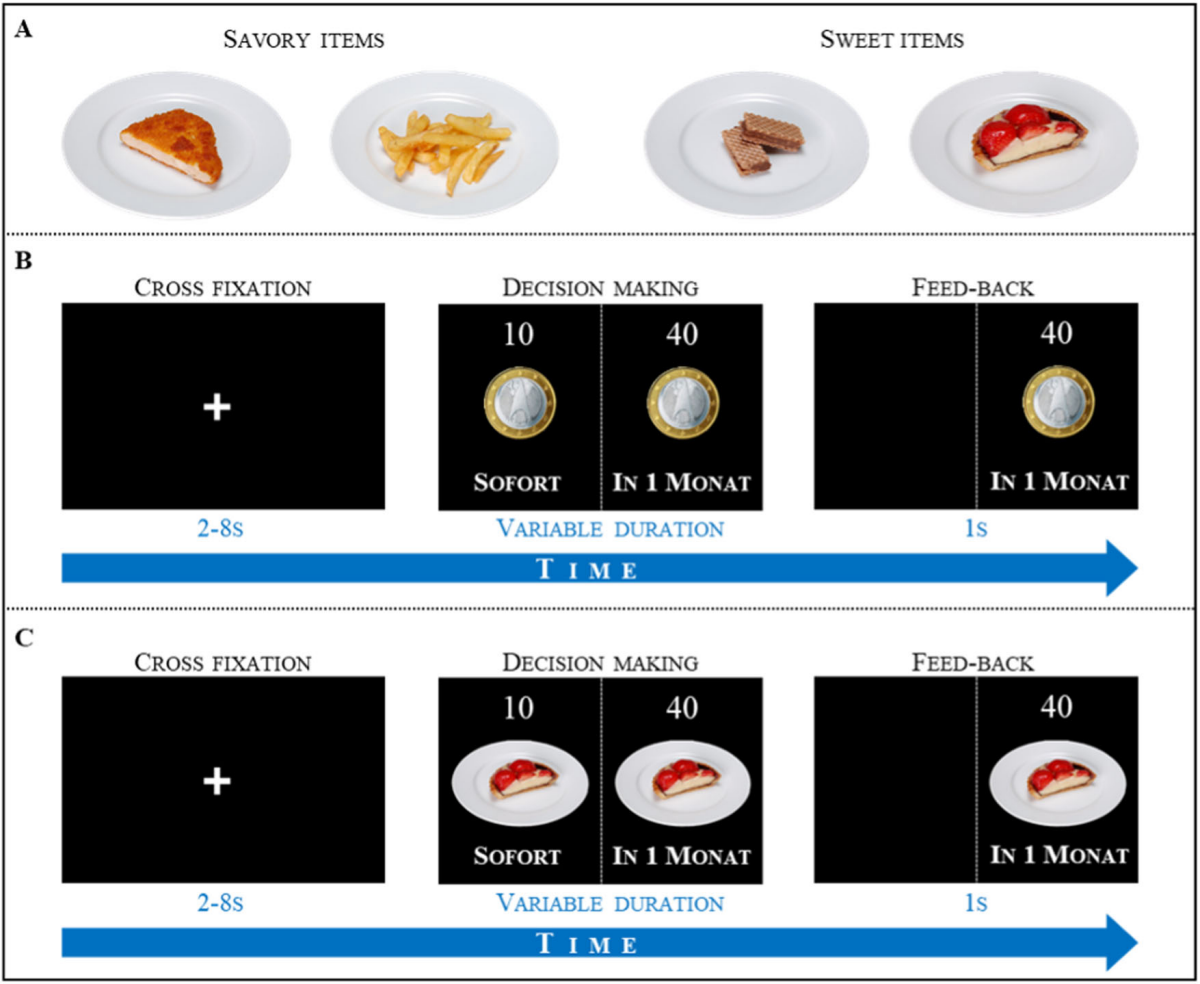
***A***, Food options presented to the participants before starting the DD task. Example of (***B***) money and (***C***) food DD task trials. During fMRI, participants had to choose between a smaller but immediate or a larger but delayed reward. In each trial, participants first saw a fixation cross in the center of the screen (2–8 s, in 1 s steps). The following presentation of the two reward options was ceased when participants pressed the button, resulting in a variable duration of the decision-making phase. The chosen option remained on the screen for 1 s before the next trial started (feedback phase). “In 1. Monat” means in one month; “Sofort” means now.

While lying in the MRI scanner, before performing the DD task, participants had to choose one preferred food item out of two savory options (schnitzel or fries) and one of two sweet options (chocolate bar or strawberry cake; Fig. 1*A*). The DD task instructions were displayed via MRI-compatible liquid-crystal dislay goggles using the Presentation® software (Version 14, Neurobehavioral Systems). Immediate and delayed options were always presented on the left and right side of the screen, respectively, to avoid any errors due to permanently changing associations between the offer and presentation side. In each of the two DD tasks (i.e., food and money), participants had to choose between an immediate and a larger but delayed reward. Delays were the following: 2 d, 2 weeks; 1, 3, and 6 months; and 1 year (Schiff et al., 2016). The amount of delayed reward always equaled 40 units. For the control (money) DD task, units were Euros (Fig. 1*B*), and for the food, DD task units were portions as displayed on the screen (Fig. 1*C*). The amount of immediate reward was determined by the amount adjustment procedure (Du et al., 2002). Accordingly, immediate reward was initially set to 20 units. If the participant chose the delayed reward, the value of the next immediate reward was systematically increased by 10 units for the second trial of the block. For the third trial, the value was increased to 5 units, for the fourth trial by 2.5 units, and for the last trial by 1 unit. If participants choose the immediate reward, the value of the immediate reward was decreased instead of increased by the same factors (i.e., 10, 5, 2.5,1 unit/s). The presentation of the two rewards was ceased when the participant pressed the button on the LumiTouch keypads (Photon Control)—either with the left or right index finger (according to the side where the chosen reward was presented; Fig. 1*B,C*). This was followed by the presentation of the feedback (for 1 s). A variable intertrial interval of 2–8 s (in 1 s steps), in which a fixation cross was presented (Fig. 1*B,C*), separated two successive trials. After participants completed five choices for a given delay, another randomized five-choice block with a different delay was presented. Each version of the DD task encompassed six blocks of five decisions each. The food and the money DD tasks were alternately performed twice (i.e., in total 4 runs of 30 trials). Task order was kept identical across participants, starting with a block of food items. All runs were ordered as follows: food–money–food–money. Participants were informed that there were no correct or incorrect choices and instructed to answer as spontaneously as possible. They were also informed that choices were hypothetical, and outcomes not delivered. This is a common procedure, since hypothetical and real rewards were shown to generate comparable behavioral responses (Madden et al., 2003; Odum, 2011).

### MRI scanner and scanning parameters

MRI measurements were performed at a 3 tesla whole-body scanner (Achieva X-series, Philips), equipped with a 32-channel head coil. For fMRI, we used a multiband echo-planar imaging (EPI) sequence with a multiband acceleration factor of 2. We acquired 38 transaxial slices parallel to the anterior–posterior commissure covering the whole brain using a voxel size of 2 × 2 × 3 mm^3^ (repetition time = 2,500 ms; time to echo = 35 ms; flip angle = 90°; field of view, 224 mm, no interslice gap). For each participant, we additionally acquired a high-resolution T1-weighted structural image with 176 transversally oriented slices covering the whole brain (isotropic T1 turbo field echo sequence: voxel size, 1 × 1 × 1 mm^3^; field of view, 240 × 176 mm^2^). These scans were used to correct for geometric distortions and perform coregistration with the EPI scans.

### Data analyses

FMRI data analyses were performed using the statistical parametric mapping software version12 (Wellcome Centre for Human Neuroimaging, University College London) running in MATLAB 2020B (MathWorks). Behavioral data were analyzed with Python (version 3.6) using the SciPy package (version 1.9.3), Nilearn (version 0.10.2), PYMC (58.1; Abril-Pla et al., 2023), and ArviZ (0.16.1; Kumar et al., 2020).

### Models of DD

We tested how five subjective value (SV) choice models and three heuristic choice models captured individuals’ discounting behavior. The five subjective choice models can be categorized along two axes: the general shape of the discounting function and whether the model included scaling or not. In all discounting models, SV is the subjective value, *A* the amount of options, and *k* a measure of impulsivity, that is, how steeply reward is devalued with increasing delay *D*. *s*, the scaling factor, varies in its function. For exponential discounting with scaling (Green and Myerson, 1996; McKerchar et al., 2009; Peters et al., 2012), *s* exponentially scales both, *k* and delay *D*, whereas for hyperbolic discounting, two scaling options exist: scaling either of delay or of the whole denominator (1 + k ∗ D). Each DD equation is applied to both immediate and delayed rewards, where the SV of an immediate reward equals its amount. See Table 1 for a detailed description of the five models.

**Table 1.** Listed are the five SV discounting models: exponential and hyperbolic discounting, either with or without second scaling

	Exponential discounting	Hyperbolic discounting
No time scaling	SV=A*exp(−kD)	$\mathrm{SV}\;=\;\;\frac{A}{\left(1\;+\;kD\right)}$
Time scaling	$\mathrm{SV}\;=\;A\;*\;\exp(-{\left(kD\right)}^{s})$	$\mathrm{SV}\;=\;\;\frac{A}{1\;+\;k\;*\;D^{s}}$ or $\mathrm{SV}\;=\;\;\frac{A}{{\left(1\;+\;kD\right)}^{s}}$

SV is the subjective value, *A* is the amount of options, *k* is the discounting factor that multiplies the value of reward, *D* is the time delay, and *k* is the degree of discounting. The scaling parameter *s* in the lower exponential discounting equation describes scaling of individual differences in delay and *k* (Loewenstein and Prelec, 1992; Rachlin, 1989; Green and Myerson, 2004; McKerchar et al., 2009; Peters et al., 2012). For hyperbolic discounting, scaling either affects just the time delay or it additionally considers *k*. Note that when *s* = 1, there is no time scaling, or in other words, the model with time scaling equals the model without scaling. When *s* is <1, the SV is more sensitive to changes at shorter delays and less sensitive to changes at longer delays (Frederick et al., 2002).

SV choice models compute independent SVs for immediate and delayed reward. Heuristic choice models, instead, are based on the idea of attribute-wise instead of option-based comparisons, where both options are first evaluated independently of each other in terms of reward amount and time delay before they are compared [SV(Delayed) − SV(Immediate); Wulff and van den Bos, 2018]. The three heuristic models we used (Table 2), can be categorized into simple (ITCH, intertemporal choice heuristic) and complex comparative functions (TRADE, trade-off model). The ITCH model assumes that four basic comparisons take place, each weighted by a subject-specific factor (Marzilli Ericson et al., 2015). The dual reasoning and implicit framework theory (DRIFT) model assumes a similar comparison for absolute reward and delay but introduces a scaling function weighting relative delay against relative reward amount (Read et al., 2013). The TRADE model specifically scales reward amount and delay by a logarithm function and a subjective scaling value (Scholten and Read, 2010). For comparing delays, the relative distance of scaled delays is multiplied by their differences, which represents the absolute relevancy of delay (factor 4 in Table 2). See Table 2 for details.

**Table 2.** Heuristic choice models (Wulff and van den Bos, 2018): The ITCH model contains four free parameters: $\beta_{xA}$ is the relevancy of absolute reward, $\beta_{xR}$ is the relevancy of relative reward, $\beta_{tA}$ is the relevancy of absolute delay, and $\beta_{tR}$ is the relevancy of relative delay

	ITCH	DRIFT	TRADE
Factor 1	$\beta_{xA}\;*\;(x_{2}-x_{1})$	$\beta_{xA}\;*\;z(x_{2}-x_{1})$	$v(x)=\left(\frac{1}{\mathrm{scalin}g_{2}}\right)\;*\;\log(1+\mathrm{scalin}g_{2}\;*\;x)$
Factor 2	$\beta_{xR}\;*\;\frac{\left(x_{2}-x_{1}\right)}{\frac{\left(x_{2}+x_{1}\right)}{2}}$	$\beta_{xR}\;*\;z\left(\frac{x_{2}-x_{1}}{x_{1}}\right)$	$\left(v(x_{2})-v(x_{1})\right)$
Factor 3	$\beta_{tA}\;*\;(t_{2}-t_{1})$	$\beta_{tA}\;*\;z(t_{2}-t_{1})$	$w(x)=\left(\frac{1}{\mathrm{scalin}g_{3}}\right)\;*\;\log(1+\mathrm{scalin}g_{3}\;*\;x)$
Factor 4	$\beta_{tR}\;*\;\frac{\left(t_{2}-t_{1}\right)}{\frac{\left(t_{2}+t_{1}\right)}{2}}$	$\beta_{xt}\;*\;z\left({\left(\frac{x_{2}}{x_{1}}\right)}^{\frac{1}{t_{2}-t_{1}}}-1\right)$	$-\mathrm{scalin}g_{1}\;*\;(w(t_{2})-w(t_{1}))$
SV(DEL)-SV(Immediate) =	Factor 1 + Factor 2 + Factor 3 + Factor 4	Factor 1 + Factor 2 + Factor 3 + Factor 4	Factor 2 − Factor 4

For the DRIFT model, $\beta_{xA}$ and $\beta_{tA}$ are equivalent to the ITCH model, but $\beta_{xR}$ is additionally scaled by the amount of immediate reward. $\beta_{xt}$ is a proportional distance factor that increases with larger differences in reward amount and decreases with larger differences in delay. TRADE utilizes a logarithmic scaling function for reward amount and delay (see Factors 1 and 3), where Scaling 2 scales both reward amounts and Scaling 3 scales absolute and relative delays. Finally, Scaling 1 scales the relative delays for their relevance.

To model choice behavior, SV equations are implemented into a choice rule that maps SV(Delayed) − SV(Immediate) to the probability of choosing either the delayed or immediate reward. In line with previous modeling attempts (Wulff and van den Bos, 2018), we used the inverse logit function with a range limited by an error value so that the output was restricted to [error, 1-error]. This error term can be interpreted as inconsistencies in discounting behavior in two ways, either for a specific delay or across delays:

$$p(\mathrm{choice})\;=\;(1-2\;*\;\mathrm{error})\;*\;\frac{\exp(\mathrm{SV}(\mathrm{Delayed})\;-\;\mathrm{SV}(\mathrm{Immediate}))}{1\;+\;\exp(\mathrm{SV}(\mathrm{Delayed})\;-\;\mathrm{SV}(\mathrm{Immediate}))}\;+\;\mathrm{error}.$$

For model comparison, we assumed normal distribution for all parameters and applied them to Monte Carlo Markov chain (MCMC) model estimation/comparison using the PYMC software (Abril-Pla et al., 2023). We defined priors by means of a uniform mean and standard distribution (see Table 3 for their respective range). All priors were defined as independent distributions for each participant.

**Table 3.** Predefined hyperpriors for parameter estimation for all parameters

SV parameter	Mu range (uniform)	SD range (uniform)	Starting point (Mu, SD)
*K* (normal)	[E^−10^ |E^−2^]	[10^−2^|3]	E^−2.5^ |1
*s*	(0|4]	[0.01|3]	1|1
Heuristic models			
$\beta_{xA}$	[−1,1]	(0,1]	0.5|0.5
$\beta_{xR}$	[−1,1]	(0,1]	0.5|0.5
$\beta_{tA}$	[−1,1]	(0,1]	0.5|0.5
$\beta_{tR}$	[−1,1]	(0,1]	0.5|0.5
$\beta_{xt}$	[−1,1]	(0,1]	0.5|0.5
$\mathrm{scalin}g_{1}$	[−1,1]	(0,1]	0.5|0.5
$\mathrm{scalin}g_{2}$	[−1,1]	(0,1]	0.5|0.5
$\mathrm{scalin}g_{3}$	[−1,1]	(0,1]	0.5|0.5
Inv-Logit			
Error	(0,0.2]	(0,0.2]	0.1|0.1

All parameters were normally distributed. Their mean (Mu) and standard deviation (SD) were uniformly distributed. Listed are the ranges of uniform distributions. Following standard range notation, round brackets indicate that the distribution does not include the given value and squared brackets indicate that the value is within the range, for example, [0, 1] would indicate a normal distribution with values larger than 0 that can include 1.

### Bayesian parameter estimation

Models were initialized using an identity matrix and then sampled using the No-U-Turn Sampler as implemented in the PYMC software (Abril-Pla et al., 2023). We initiated the MCMC process with 60,000 iterations as a burn-in phase, after which we collected 1,500 samples from the posterior distribution. Less samples in the burn-in period led to Rhat scores larger than 1.1, indicating significant difference between chains and hence less reliable parameter estimates (Vehtari et al., 2021). To compare model fit between both conditions (i.e., money and food discounting), the sampler was informed about individuals’ behavior in one of the two conditions. To identify which model best captures choice behavior, we used the Watanabe-Akaike Information Criterion (WAIC; Watanabe, 2013) as implemented in the ArviZ 0.16.1 software (Kumar et al., 2020), and the negative-log scale where optimal model fit is indicated by low WAIC values.

### Parameter recovery

For parameter recovery, we used the estimated parameters of the winning model, which was exponential discounting with scaling for money and food, and simulated real choice behavior using the estimates from our previous model fit. Afterward, we used the same method to estimate model parameters. Using the means across samples, we found significant correlations between real and estimated parameters for *k* (*r* = 0.66; *p* < 0.05), but not for *s* (*r* = −0.275; *p* = 0.173) or the error term (*r* = −0.1347; *p* = 0.511), which renders differences in parameter estimates for *s* between food and money nonreliable.

### Condition effect

To compare the influence of the condition (food or money) on discounting behavior, we used the delta of parameter estimates for *k* between conditions and calculated their 95% highest density interval (HDI).

### fMRI data processing

FMRI data preprocessing included slice time correction, realignment to the first image of the time series, normalization of images applying the parameters from T1 normalization, and smoothing with an isotropic Full-Width at Half-Maximum (FWHM) filter of 7 mm. Preprocessed images were analyzed with the general linear model. To assess the main effects of money and food rewards, we modeled each participant's data with two regressors, respectively. We modeled neural activity related to the evaluation and decision-making phase using a stick function, placed on the onset of the presentation of the two offers, convolved with the hemodynamic response function. Feedback events were modeled with an additional regressor. To minimize false-positive activations due to task-correlated motion, we also included the six head motion parameters as regressors of no interest. We applied a high-pass filter at 128 Hz. Serial correlations were accounted for by first-order autoregression [i.e., AR(1)]. Model parameters were then estimated using the restricted maximum likelihood method. Signed *t*-contrasts (i.e., +1) were applied to each of the two regressors, to assess the effects for food and money discounting, respectively. For the group-level analysis, the two first-level contrast images were applied to a paired *t* test. Clusters were considered significant if they survived a peak voxel threshold of *p* < 0.05, false discovery rate (FDR) across the entire brain volume.

## Results

### BIS-15

Participants’ BIS-15 scores indexing impulsivity (total mean = 33.07 ± 6.6) were comparable to those reported, for example, by Orozco-Cabal et al., (2010) in healthy individuals. BIS-15 total scores and its subscales did not correlate with *k* for both conditions (for food: *k*∼Total, *p* = 0.48, *r* = −0.14; *k*∼Nonplanning, *p* = 0.53, *r* = −0.12; *k*∼Motor, *p* = 0.34, *r* = −0.19; *k*∼Attentional, *p* = 0.35, *r* = −0.18; for money: *k*∼Total, *p* = 0.57, *r* = 0.133; *k*∼Nonplanning, *p* = 0.51, *r* = 0.135; *k*∼Motor, *p* = 0.79, *r* = −0.05; *k*∼Attentional, *p* = 0.50, *r* = 0.135).

### Modeling of discounting behavior

**Figure 2.**
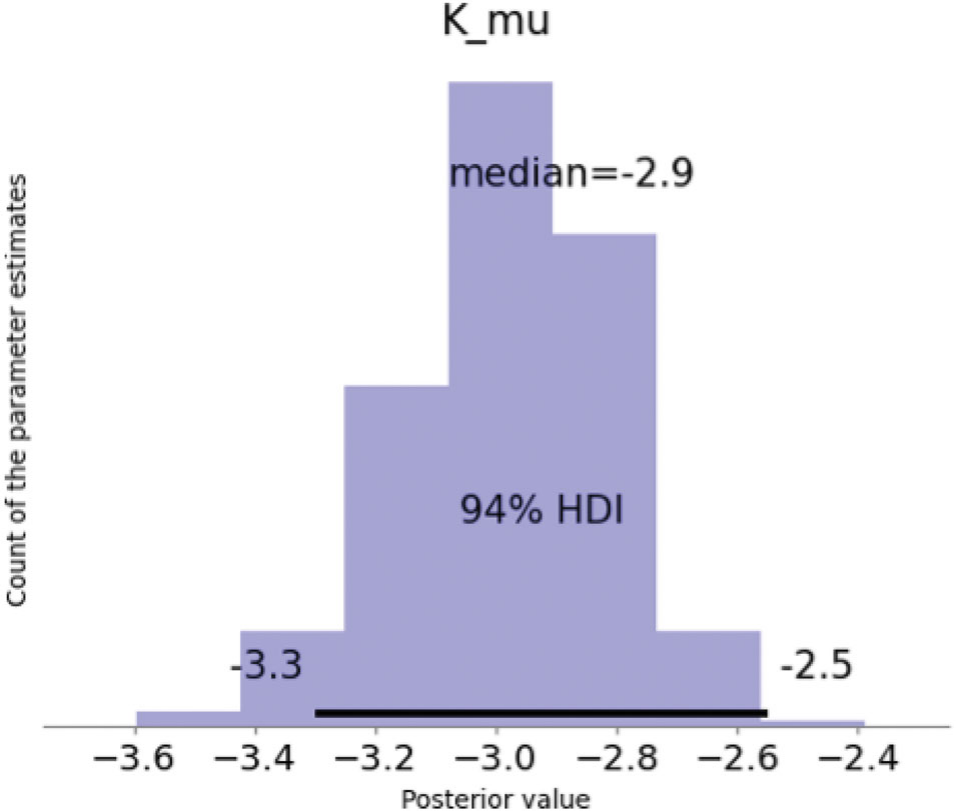
Plot of the posterior distribution of the hyperpriors *k*_Mu (Table 3) for the exponential discounting model with scaling in the food condition. The *y*-axis tracks the relative count of the parameter estimates, and the *x*-axis the estimated parameter value. The assumed normal distribution is not cutoff at the right side, which would have affected our sampling.

**Figure 3.**
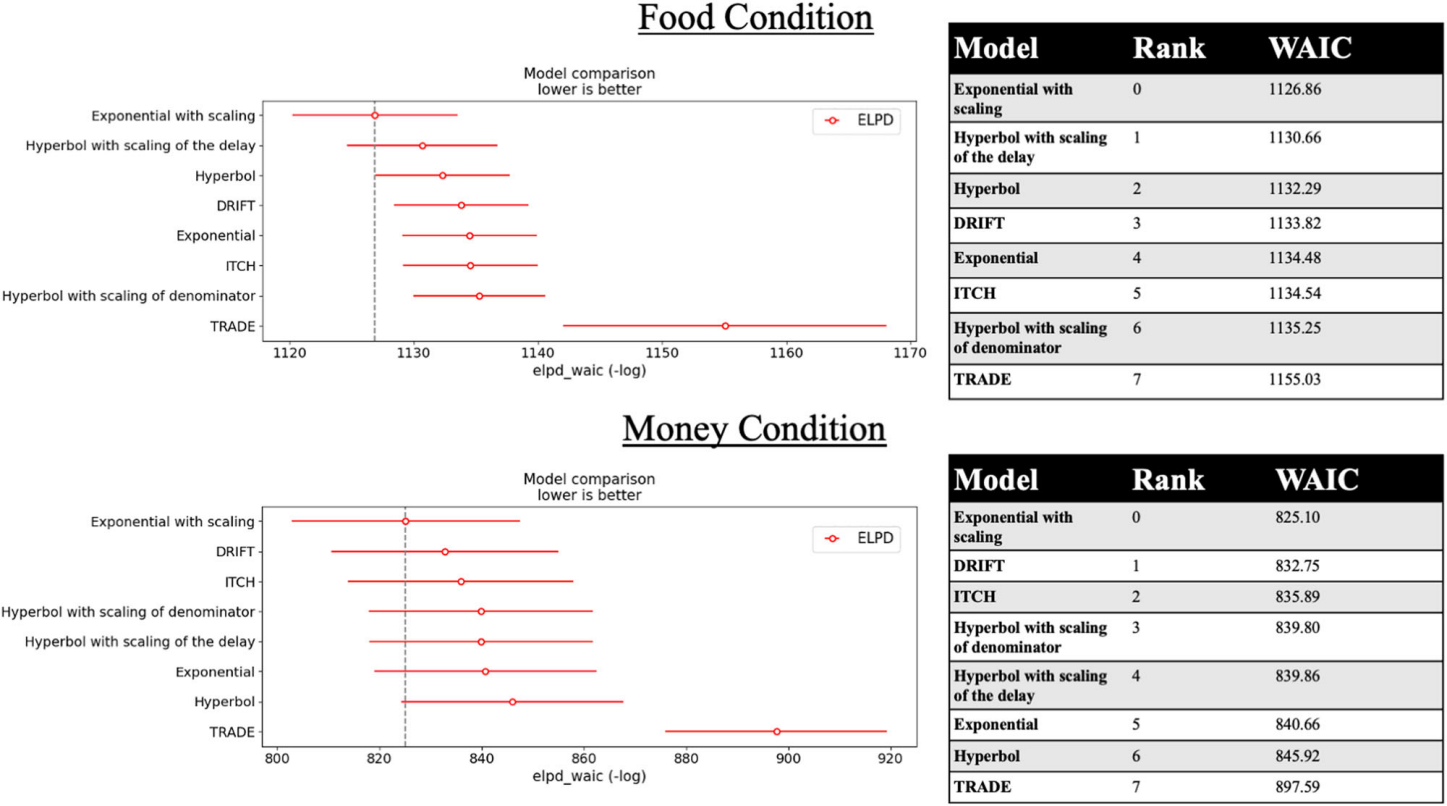
Model comparison for food and money discounting. In the plots on the left, red circles indicate WAIC scores (scaled as the negative log-likelihood), and the red line indicates standard deviations. The tables on the right list model-wise absolute WAIC scores.

WAIC revealed that the discounting of both rewards, food and money, was best captured by exponential discounting with scaling, which, for both reward types, captured behavior significantly better than the next best-fitting models. The posterior distribution of *k*_Mu (Table 3) was sampled from a normal distribution close to 0. This assumed normal distribution was not cut off at the right side (Fig. 2), which would have affected our sampling. Hyperbolic discounting with scaling of the denominator, our expected winner for the money condition, was the second best-fitting model for food and the fourth best-fitting model for money. See Figure 3 for more details.

### The influence of reward type

**Figure 4.**
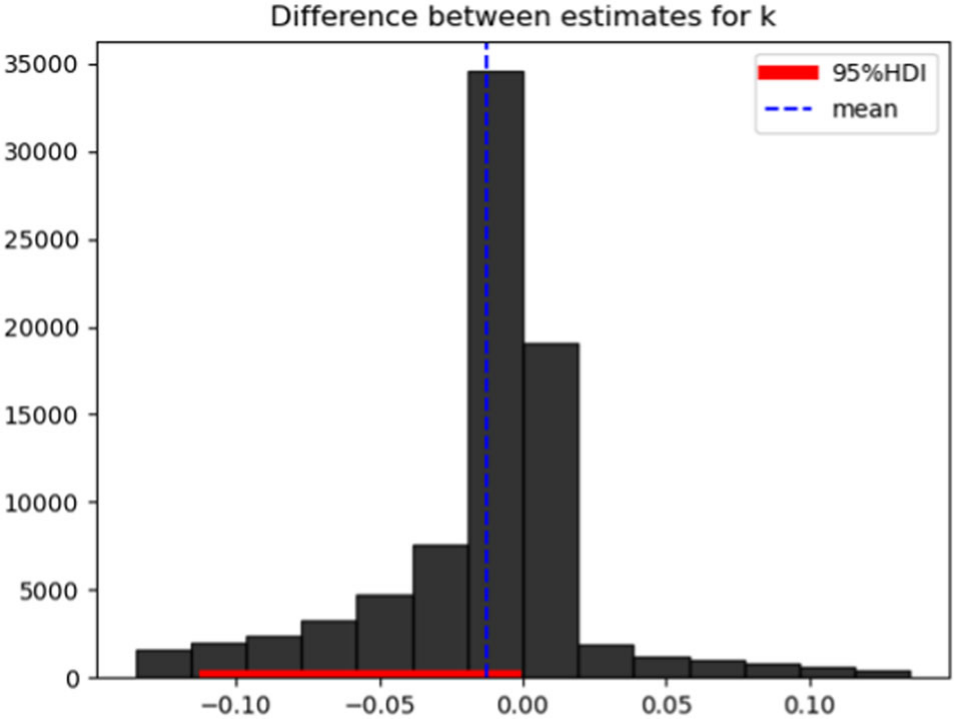
Estimates for the difference in *k* (*k*_food_ − *k*_money_) between conditions. The 95% HDI is in the range [−0.1, 0] with a mean difference of −0.01259, indicating steeper discounting of food rewards.

Since discounting behavior for food and money was best described by exponential discounting, we next compared parameter estimates for *k* not *s* due to the non-significant parameter recovery. The mean difference between conditions was −0.01259 with an HDI of [−0.1, 0], indicating steeper discounting of food than money rewards (Fig. 4). We also tested whether *k* values for food and money discounting are correlated with each other but found no significant association (*r* = −0.09; *p* = 0.65).

### Differences in brain activation between money and food discounting

**Figure 5.**
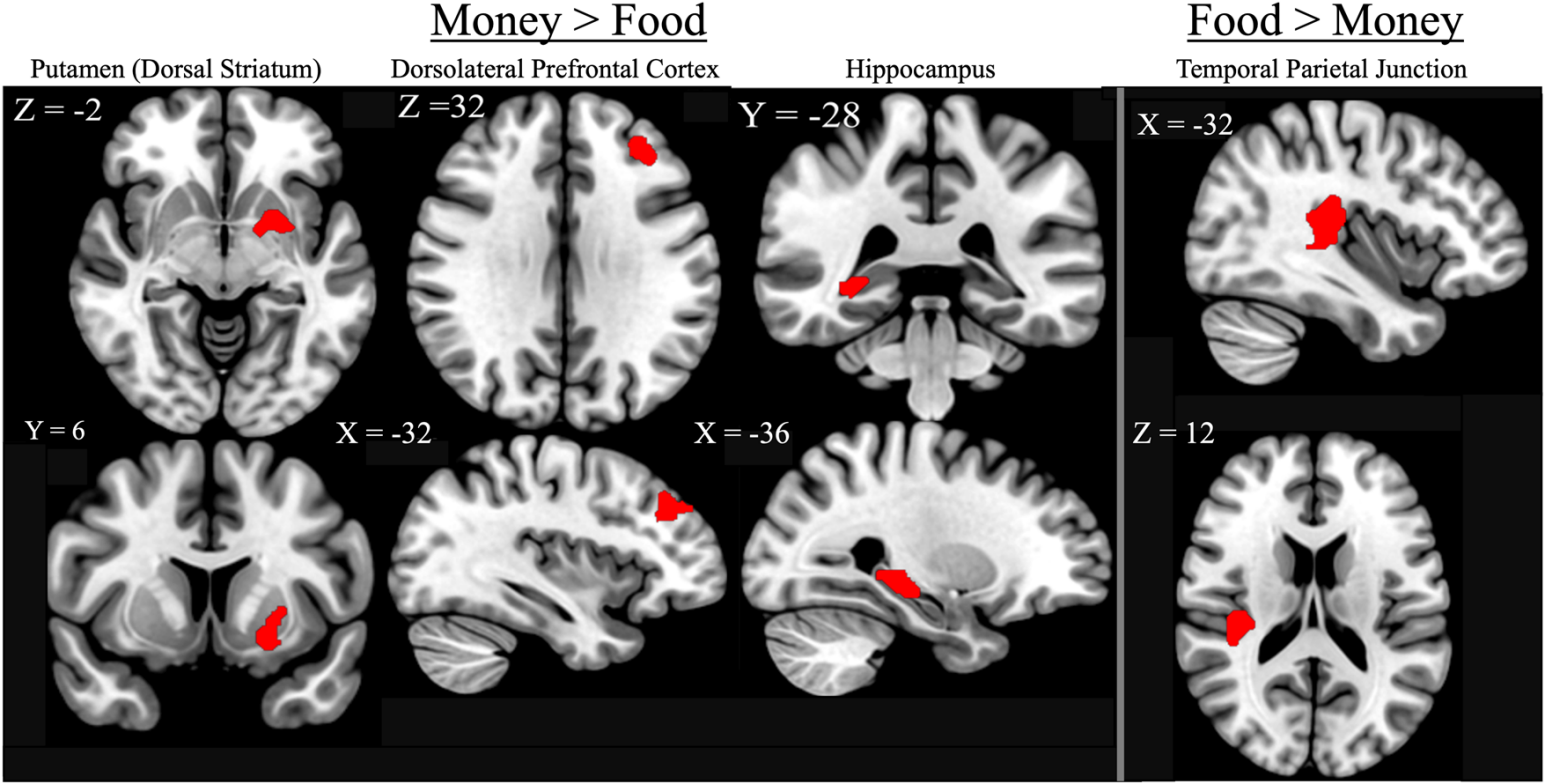
Condition-specific brain activity. Money as compared with food discounting revealed significantly [i.e., *p*(FDR) < 0.05] enhanced activity in the right putamen, (i.e., dorsal striatum), right dorsolateral prefrontal cortex, and left hippocampus. For food as compared with money discounting, we found enhanced activity only in the left temporoparietal junction. For *x*, *y*, and *z* coordinates, cluster size (amount of activated voxels), and *T*-scores, please refer to Table 4.

Comparing money with food discounting revealed significant activity in the right dlPFC, left hippocampus, and right putamen (i.e., dorsal striatum). For food as compared with money discounting, we identified only one significantly activated region, namely, the left temporoparietal junction (Fig. 5). See Table 4 for the topographic assignment of brain activations, corresponding *x*, *y*, and *z* coordinates, cluster sizes (amount of activated voxels), and the *T*-scores.

**Table 4.** Regions with significant higher activity for money as compared with food and vice versa

Name	*x*	*y*	*z*	*k*(cluster size)	*T*
Money > Food					
Hippocampus	−36	−22	−18	296	4.21
dlPFC	−32	34	36	912	4.06
Putamen	32	2	2	2,216	3.69
Food > Money					
TPJ	−32	−30	12	256	3.91

The table lists regions’ topographic assignment, coordinates in MNI space, cluster size (i.e., amount of activated voxels), and the *T* values. dlPFC, dorsolateral prefronal cortex; TPJ, temporoparietal junction.

## Discussion

The objective of our study was to identify differences in discounting behavior and related brain responses for different types of reward. To this end, we questioned whether humans deploy different strategies for discounting primary (i.e., food) or secondary rewards (i.e., money).

According to our a priori hypotheses, we found that a rather simple option-based computational model (i.e., exponential discounting) captures the “now-or-later” food/money decisions of our task better than more complex attribute-wise models. These latter heuristic models consist of a larger parameter space (compare Tables 1, 2) and hence allow to describe distinct behavior features more precisely. On the other hand, larger complexity is penalized by Bayesian model comparison, such as WAIC, which suggests that results of our model comparison were driven by both model sensitivity and model complexity. Scaling of *k*, as a measure of impulsivity, together with the delay between rewards, had a decisive influence on the model's superiority, since exponential discounting alone was outperformed by many other, not only option-based, alternatives. These findings underscore the importance of considering scaling of individual differences in time insensitivity/sensitivity to reliably capture superior features of discounting behavior with Bayesian modeling (Ebert and Prelec, 2007). Schüller et al. (2021) have chosen a comparable approach and found that discounting money over shorter delays (up to 6 months compared with 1 year in our study) is also better captured by exponential as compared with hyperbolic discounting, which supports the validity of our findings even though our task included exceptionally long delays (e.g., 6 and 12 months). Despite convincing results of our model comparison, the parameter recovery of the winning model was only successful for *k*, as a measure of impulsivity, but not s, a factor of time sensitivity. These findings question the latter parameter's reliability and, hence, its interpretability. *k*, however, could be reliably recovered, suggesting that a simple exponential function with scaling is well suited to reliably capture the degree of impulsivity during discounting.

**Figure 6.**
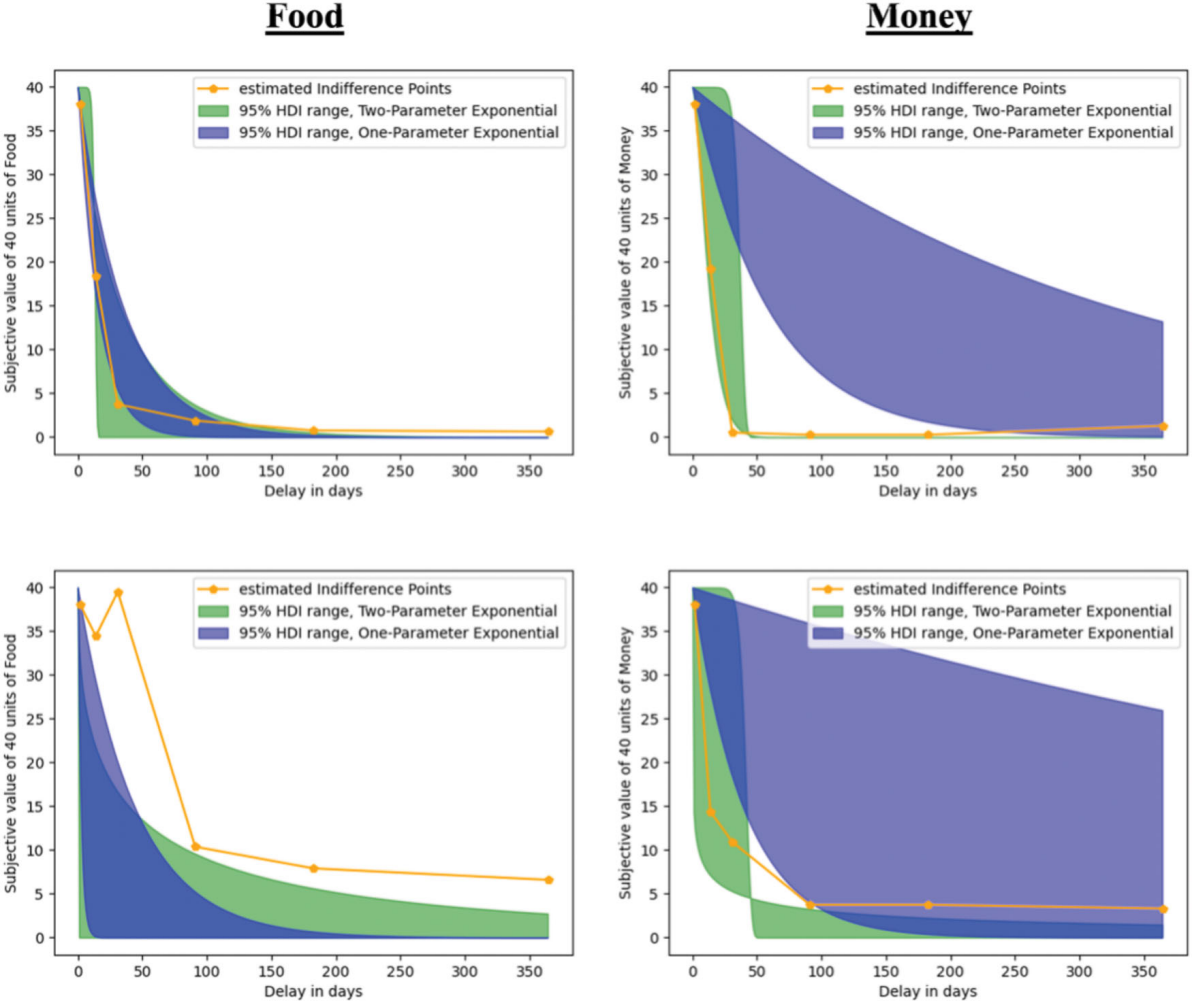
Exemplary plot for two participants, a typical impulsive one with high *k* values (top row) and a less impulsive one with low *k* values (bottom row), showing the estimated indifference points (orange), the 95% HDI range of estimated parameters for the two-parameter exponential discounting model (green), and the one-parameter version (blue). The *y*-axis shows the estimated SVs for 40 units of food (2 plots on the left side) and money (right side). The *x*-axis shows the delay in days. The plots show that, even with varying levels of discounting, the two-parameter model better aligns with the estimated indifference points than the one-parameter model.

As compared with our study, the parameter recovery using DD models often demonstrates substantially higher correlation coefficients, such as 0.85–0.98 (Ballard et al., 2023) or even 0.90 and 0.95 (Wagner et al., 2020, 2023) for single-parameter models. Our correlation coefficient of 0.66, while acceptable, appears noticeably inferior and may be influenced by the scaling parameter *s*, so that *k* is reliable but impacted by the unreliability of *s*. The exponential DD model, especially if compared with more complex models, may lead to robust parameter recovery but could sacrifice some model fit. Simpler models are often preferred because they have fewer parameters and therefore less parameter interactions, making it easier to estimate them more accurately. As compared with more complex models, they may, however, not capture all the nuances of individual behavior. Figure 6 illustrates how estimated indifference points align with the winning exponential discounting model. The plots show the one-parameter model (depicted in blue) and the two-parameter model (represented in green), which includes the scaling factor *s*. With this figure, we show parameter estimates for both a typical impulsive participant with high *k*-values (top row) and a less impulsive one with low *k*-values (bottom row). Irrespective of the conditions (food and money), the degree of impulsivity, and the various delays considered, individual parameter estimates exhibit a superior fit to the two-parameter exponential model as compared with the one-parameter version. This illustrates that the inclusion of the scaling parameter *s* in our study carries significance and improves model fit across both conditions. This observation also counters the possibility that the modest parameter recovery (0.66) indicates that the model only accurately represents individuals’ discounting behavior for specific delays and not all.

For primary reward (i.e., food), we found the expected constant loss of reward value over time, most likely due to the generally limited durability of food. For secondary reward (i.e., money), we contrarily expected significant weaker flattening of SV at longer delays due to the long-term stability of the value of money, best explained by hyperbolic discounting. Our results tend to reflect these different behavioral patterns for monetary and food rewards, which, however, were not different enough to be explained by different behavioral models. Significant differences in *k* between food and money discounting suggest that individuals generally act more impulsive in the face of primary than secondary rewards (De Petrillo et al., 2020; Odum et al., 2020).

One potential explanation for higher impulsivity during food discounting lies in our evolutionary history. Throughout human evolution, access to food was often uncertain, and the ability to obtain sustenance had clear survival advantages (Nettle et al., 2017). This historical context may have led to a stronger preference for food rewards and a higher *k* value for food discounting. Biologically, our brains are moreover wired to prioritize food as a primary reward (Sescousse et al., 2013; Simon et al., 2015; May-Wilson et al., 2022; Oren et al., 2022). The brain's reward system is highly responsive to food cues that offer gratification of basic needs such as hunger and thirst. This satisfactory aspect can lead individuals to place a higher affective value on food rewards. Money, on the other hand, is a more abstract and versatile resource that can be saved, invested, or used for a wide range of purposes over time (Simon et al., 2015). Physiologically, food, even if presented only visually, engages sensory associations, including taste and smell, which together make food highly desirable (Oren et al., 2022), contributing to a higher *k* value. Money lacks these sensory attributes and is less inherently rewarding on a sensory level. Together, these evolutionary, biological, and sensory factors will shape more impulsive behavior if individuals are confronted with food instead of money reward. This underscores the importance of considering various influences when examining discounting behavior in different contexts.

There are several limitations that warrant careful consideration when interpreting our findings. Firstly, the prices of the food items presented did not correspond to the typical prices in German supermarkets. A more significant limitation lies in the inclusion of trials with exceptionally long delays (e.g., 6 and 12 months) and substantial reward quantities, particularly in the context of food items (e.g., 40 pieces of cake). Despite the long delays, savory foods were presented fried (schnitzel) and deep-fried (fries), which additionally raises concerns about the ecological validity of our results. To address this concern, we conducted additional analyses after excluding food and monetary trials with payoffs occurring after 6 and 12 months. Although these supplementary analyses confirmed the results of previous studies (Schüller et al., 2021) and our results obtained from the complete dataset, it remains uncertain whether participant behavior would have differed, had they been confronted with individually tailored food items and more realistic prices. Especially for highly delayed rewards, higher-priced food is more likely to be discounted than lower-priced food. Achieving this individualization could have been accomplished by querying participants about how much they would have been willing to pay for specific food items. In future studies, it is advisable to consider using individualized reward amounts (Odum and Rainaud, 2003), equitably distributed in terms of value among reward types (Estle et al., 2007), as this may enhance the sensitivity of detecting differences in the discounting of primary and secondary rewards. Food items should not be fried or deep-fried as this suggests immediate consumption. Future task design should also consider offering food items for vegetarians or vegans since avoiding meat or fish becomes increasingly popular. Another limitation is the fixed task design (food–money–food–money), which may have caused order effects. To avoid such confounding influences, future studies should consider a balanced design across participants (i.e., half of participants, money–food–money–food; other half, food–money–food–money) which would allow to assess systematic errors due to the trial order. The last limitation we discuss pertains to the use of hypothetical rewards in our study. While previous research has demonstrated that hypothetical and actual rewards can yield similar behavioral responses (Madden et al., 2003; Odum, 2011), it is common practice to employ real outcomes for a single random decision. Whether genuine monetary and food rewards induce similar behavioral patterns, as those shown in the present study, remains an open question for future research.

For money as compared with food discounting, we identified the expected enhanced brain activity in the dlPFC, involved in executive control (Funahashi, 2001; Wagner et al., 2001), but neither in the vmPFC nor the ACC. Non-significant findings should be interpreted with caution since they still may become significant with increasing the sample size, but a lack of reward-related differences in the vmPFC and ACC could rely on similar processing demands for both reward types in terms of value coding and choice difficulty, respectively. The dlPFC, particularly in the right hemisphere, plays critical roles in the avoidance of risky choices (Obeso et al., 2021), the difficulty in discounting money rewards (Jimura et al., 2018), and the framing of gains and losses during intertemporal decision-making (Xiong et al., 2023). Money is a strong motivator and associated decisions often require more cognitive effort and self-control (Lea and Webley, 2006), leading to the heightened dlPFC activity for money as compared with food reward as individuals contemplate the long-term benefits of delaying gratification (Beck et al., 2010).

The hippocampus is involved in episodic memory processing (Bird and Burgess, 2008) and, hence, memory-demanding reward-based decision-making (Wimmer et al., 2014). In the context of money discounting, individuals may rely more on episodic memory and future planning, thus engaging the hippocampus (Guo et al., 2022; Schacter et al., 2017), whereas food choices are influenced more by sensory and immediate experiences (Gibson, 2006), probably requiring less hippocampal involvement.

Besides enhanced activity in right dlPFC and left hippocampus, we also observed money discounting-related enhanced activity in the right ventral putamen. The putamen, together with the nucleus caudatus, constitutes the dorsal striatum, which is linked to habit formation (Malvaez and Wassum, 2018; Baker et al., 2023) and long-term inferences (Fischer et al., 2017). In line with these findings, the structural and functional connectivity between the striatum and dlPFC during DD was found to be associated with less impulsivity and increased patience (Van Den Bos et al., 2014). In our study, enhanced striatal responses for money discounting may relate to more demanding inferences in the face of attractive money gains. In contrast, food choices may rely on more affective responses, resulting in less dorsal striatum activation.

Comparing food discounting with money discounting revealed significantly enhanced activity in only one brain region, namely, the temporoparietal junction (TPJ), which is involved in reasoning beliefs of others (Samson et al., 2004; Schurz et al., 2013), social perspective taking (van den Bos et al., 2010), and making sense of another mind (Saxe and Wexler, 2005). In many societies, sharing food is a common practice, and food often serves as a symbol of hospitality and community (Hanna et al., 2023). In the context of larger food amounts, individuals in our study might have considered sharing food with others, which includes considering others’ preferences and needs. This social reinforcement of the value of food rewards may have contributed to higher *k* values in food discounting and engaged left TPJ as individuals weighted social aspects for food choices stronger than for money choices.

## Conclusions

In summary, our findings reinforce the assumption that humans deploy common behavioral strategies for the discounting of primary and secondary rewards. In persons with addictive behaviors, self-control over primary rewards is a critical issue. Future studies should investigate primary versus secondary reward-specific discounting behavior to determine the general or specific nature of poor self-control in this population. Uncovering the behavioral facets specifically involved in self-controlled choices for primary rewards, and the extent to which they translate to other types of reward may in the future pave the way for the development of novel behavioral−–therapeutic interventions.

## Data Availability Statement

Due to ethical restrictions, the behavioral and fMRI data can only be made available on request.

## Synthesis

Reviewing Editor: Alexander Soutschek, Ludwig-Maximilians-Universitat Munchen Decisions are customarily a result of the Reviewing Editor and the peer reviewers coming together and discussing their recommendations until a consensus is reached. When revisions are invited, a fact-based synthesis statement explaining their decision and outlining what is needed to prepare a revision will be listed below. The following reviewer(s) agreed to reveal their identity: Ben Wagner, Amy Odum.

REVIEWER #1

The authors utilized functional Magnetic Resonance Imaging (fMRI) in conjunction with a delay-discounting (DD) paradigm to examine the devaluation processes of primary (food) and secondary (money) rewards in 28 healthy, normal-weight participants, selected from an initial pool of 37. By employing a blend of statistical methodologies, the researchers quantify the discounting behavior for both types of rewards. Their findings revealed that the value of money diminished at a slower pace (optimally explained by a hyperbolic model variant) as compared to food (which was optimally explained by an exponential model variant). Moreover, fMRI analyses highlighted that these variations were linked to heightened activity in the anterior insula - a brain area associated with taste perception and the formation of emotions related to the body's state of equilibrium.

Overall, the subject of this paper is compelling due to the partially unresolved question surrounding how different types of rewards are devalued over time. Specifically, it seeks to ascertain which models/functions most accurately represent the devaluation process, and whether particular brain regions play a role in modulating this process. The paper demonstrates that primary and secondary rewards -in their paradigms- undergo different discounting processes and indicates that the insula plays a part in processing or discounting food. This can constitute a valuable contribution to the field. However, I have concerns in terms of the ecological validity of the food discounting task, as well as the comprehensive statistical analysis and model comparison. Given that the authors possess control over the latter, I would recommend them to explicitly outline the limitations linked to the task and to devote more effort towards substantiating their modeling assertions. This could be achieved by adopting a Bayesian framework or adjusting their current approach.

1. The authors' statistical analysis aligns with the methods employed in Peters et al. (2012) and earlier studies. However, it's important to note that computational modeling has made substantial progress over the past decade. Many contemporary studies utilize hierarchical Bayesian models to directly estimate latent parameters (such as k, beta, or scaling). While this isn't inherently problematic - the authors indeed used a valid approach to quantify discounting - potential issues might arise in relation to the model comparison and the derived conclusions. I will put forward two suggestions for either revising the analysis or supplementing the existing methodology

New analysis to replace their approach:

2. Using a full Bayesian framework the authors could utilize improved model comparison techniques based on the entire posterior of inferred parameters of interest, such as the Watanabe-Akaike Information Criterion (WAIC) or Expected Log Predictive Density (ELPD) (Vehtari et al., 2017). The Akaike Information Criterion (AIC) used for model comparison is known to be less conservative and tends to favor overly complex models, particularly with small datasets. Alternative methods would more efficiently penalize high correlations in s and k as they account for the variability within and correlation between these parameters.

3. The authors could directly test if the scaling hyperparameter significantly deviates from 1. This would be the case if, for instance, the 95% Highest Density Interval (HDI) of the posterior of the scaling hyperparameter doesn't intersect with 1. Did the scaling parameter differ from 1 in their analysis?

4. A potential reason why models with a scaling factor are not used in recent studies is their high negative correlation with the discount rate. Consequently, the data can be equally explained by changing s or k, and as a result, the discount rate becomes unreliable. Could the authors argue why this should not matter?

5. If the authors aim to prove the superiority of such models, I believe relying solely on R2 (which overlooks complexity) and AIC is insufficient. They should provide other measures of model comparison (preferably WAIC or ELPD, as stated above) and offer posterior predictive checks to illustrate which data patterns benefit from the scaling factor. Additionally, it would be highly beneficial to demonstrate a parameter recovery to determine if s and k can be reclaimed. By doing so, the authors could strengthen their claim and enhance the value of the paper. Currently, it's unclear if the winning model genuinely reproduces crucial data patterns more accurately than others. Also, using AIC with such marginal differences does not conclusively indicate whether one model is significantly superior. The lower delta AIC could be influenced by a few participants. Code for such models is readily available online and can be effortlessly implemented in Python (using Pyro, PyTorch, STAN), R (using JAGS, STAN), or MATLAB (using JAGS, STAN). Furthermore, model code for softmax discounting models is frequently available in recent publications.

6. Such an analysis would also be beneficial as it would obviate the need to exclude participants with systematic shifts. In my experience, the exclusion rate of approximately 25% of participants in a DD paradigm is relatively high.

As an alternative, because such a completely new analysis might be an overkill and not per se justified, I would recommend expanding the existing analysis in the following ways:

1. Given the criticism of AIC for its tendency to favor complexity, I would advise the authors to at least employ other comparison methods such as the Bayesian Information Criterion (BIC) and/or Bayesian model selection. These can be computed from their analysis as demonstrated by Peters et al. (2012).

2. It would be beneficial if the authors could provide a parameter recovery for s and k for the winning models in their analysis. This would demonstrate whether the parameters are reliable or not, as suggested by modeling good practices see Wilson and Collins (2019). If these prove unreliable, the authors should openly discuss this along with the pros and cons of utilizing a scaling parameter in discounting models. Questions that could be addressed include: Is it necessary to report both measures in the future? How would one compare discounting across different studies, participant groups, or types of rewards if it is the case that discount rate is unreliable?

3. Concerning participant exclusion: The authors could normalize the indifference points so that a value of 1 signifies a consistent preference for one option (e.g., immediate or delayed rewards), and a value of 0 signifies a consistent preference for the alternative option. This normalization could help quantify discounting in participants where ID points are hard to compute because of systematic shifts. Systematically (depending on degree) high or low discounting should not be the sole criterion for exclusion. E.g. do these participants also show unusually short response times?

4. Some recent publications suggests that reward magnitude and delay are compared separately. This might also be a valuable addition to the authors model comparison.

From my perspective, there are only a few scientific tasks in the domain of value-based decision-making that function effectively and as anticipated. That being said I have reservations about the ecological validity of the food discounting task employed in this study.

1. In their abstract, the authors assert that rewards are valued equally. However, this claim doesn't appear to be supported by the task utilized. For instance, the authors equate the value of 40 steaks to 40 euros for every participant. While this is an approach they can take, it doesn't permit them to claim equal or matched value, or even subjective value (which the authors reference in terms of a 'common currency') in units. The authors could have either adopted a stepwise procedure to estimate the subjective value of a food item for each participant or at least queried participants on how much they would be willing to pay for a portion of steak or fries. Moreover, these values don't align with the real prices encountered in German supermarkets. As the authors can't rectify this, I recommend they modify their wording and more explicitly acknowledge this as a limitation. I don't believe that analyzing a subset of data addresses these issues adequately.

2. The authors suggest that hypothetical and real monetary rewards are discounted equally. While I concur with this, it's common practice for many studies to payout one random decision, and in my experience, data appears less noisy and presents fewer systematic shifts when this is done. Even though it's valid to use hypothetical monetary rewards, less is known regarding food rewards. Can the authors provide any evidence supporting an equality in discounting food? If not, this could be another limitation that warrants proper discussion.

3. There may be an issue in presenting only two main options. For vegan or vegetarian participants, only one option was available. Also, all items are cooked. It's a bit unusual to be choosing between e.g. ten portions of ready-to-eat fries today and forty portions next week. I regret to emphasize this point, as the authors can't alter this at this stage, but I believe it's imperative for the entire field to invest more time in task construction and strive for ecological validity. This is a major imitation.

I also have a few minor suggestions:

1. The authors initiate their paper by questioning whether behavior varies between discounting food and secondary rewards. Wouldn't it be insightful to present the within-participant correlation of discount rates (k) for food and money discounting for all participants? Despite the best-explaining models being different, this correlation could be informative.

2. Do the discount rates, for the best-fitting models, correlate with the BIS scores?

3. The authors propose that food should be exponentially discounted. While this is logically sound to me, I recommend providing at least some supporting evidence for this hypothesis. Are there other studies demonstrating this? Certain literature suggests that exponential models explain data from experiential discounting tasks (shorter timescale) more efficiently than hyperbolic models, for example, Johnson (2012) and Schüller et al. (2021). If food is more heavily discounted (but only within a shorter timeframe), this would be coherent.

4. The authors write, "The larger b, the greater is the inconsistency in participants' choice behavior across trials with the same reward values and delays." Theoretically, b can also increase if participants make inconsistent choices across different delays.

5. Some of the citations in the paper can be updated. There are more recent reviews from 2018 to 2022 that, for instance, summarize findings on delay discounting and psychiatric conditions, including addiction and others (see Amlung et al. 2019; Bickel et al. 2019; Lempert et al. 2019). E.g. for them motivation of the study in the introduction.

6. Could the authors clarify the exclusion criteria for the two participants exhibiting long response times in some trials? How many trials exceeded which threshold, and why was this criterion used?

Publication bibliography

Amlung, Michael; Marsden, Emma; Holshausen, Katherine; Morris, Vanessa; Patel, Herry; Vedelago, Lana et al. (2019): Delay Discounting as a Transdiagnostic Process in Psychiatric Disorders: A Meta-analysis. In JAMA psychiatry 76 (11), pp. 1176-1186. DOI: 10.1001/jamapsychiatry.2019.2102.

Bickel, Warren K.; Athamneh, Liqa N.; Basso, Julia C.; Mellis, Alexandra M.; DeHart, William B.; Craft, William H.; Pope, Derek (2019): Excessive discounting of delayed reinforcers as a trans-disease process: Update on the state of the science. In Current opinion in psychology 30, pp. 59-64. DOI: 10.1016/j.copsyc.2019.01.005.

Johnson, Matthew W. (2012): An efficient operant choice procedure for assessing delay discounting in humans: initial validation in cocaine-dependent and control individuals. In Experimental and clinical psychopharmacology 20 (3), pp. 191-204. DOI: 10.1037/a0027088.

Lempert, Karolina M.; Steinglass, Joanna E.; Pinto, Anthony; Kable, Joseph W.; Simpson, Helen Blair (2019): Can delay discounting deliver on the promise of RDoC? In Psychological medicine 49 (2), pp. 190-199. DOI: 10.1017/S0033291718001770.

Peters, Jan; Miedl, Stephan Franz; Büchel, Christian (2012): Formal comparison of dual-parameter temporal discounting models in controls and pathological gamblers. In PloS one 7 (11), e47225. DOI: 10.1371/journal.pone.0047225.

Schüller, Canan Beate; Wagner, Ben Jonathan; Schüller, Thomas; Baldermann, Juan Carlos; Huys, Daniel; Kerner Auch Koerner, Julia et al. (2021): Temporal discounting in adolescents and adults with Tourette syndrome. In PloS one 16 (6), e0253620. DOI: 10.1371/journal.pone.0253620.

Vehtari, Aki; Gelman, Andrew; Gabry, Jonah (2017): Practical Bayesian model evaluation using leave-one-out cross-validation and WAIC. In Stat Comput 27 (5), pp. 1413-1432. DOI: 10.1007/s11222-016-9696-4.

Wilson, Robert C.; Collins, Anne Ge (2019): Ten simple rules for the computational modeling of behavioral data. In eLife 8. DOI: 10.7554/eLife.49547.

REVIEWER #2

This is an interesting manuscript examining delay discounting of monetary and food rewards along with corresponding fMRI analyses. The authors maintain that food rewards activate the anterior insula more so than monetary ones. Perhaps as a result, monetary discounting is best characterized by a hyperbolic function, as has been found many times before, but food discounting is best described by an exponential function. I have a few comments for the authors to consider.

The manuscript is generally well prepared and executed. There are a few things that could benefit from some more development and/or different or additional presentation.

Introduction:

Line 68: Why is this predicted?

Participants:

Line 93-95: Did the exclusion of these participants affect the results? Ideally the authors would deposit all of the data, including from excluded participants, into a data repository like Open Science Framework (OSF) and provide a link to the data in the manuscript.

How were the participants recruited?

Methods:

Delay discounting task: The value of the monetary and food rewards may have differed at no delay. That is, they may not have been worth the same. For example, 40 units of schnitzel could be worth more than 40 Euros. Why was this technique used? It would be good to provide a citation, which would be the Estle et al. (2007) study I believe. This issue is something that would be good to address in the discussion. How would this difference be predicted to influence the outcome? If schnitzel were worth more, for example, then it would actually be predicted to be discounted less rather than more than 40 euros, based on the magnitude effect. Furthermore, in experiments when the two values are more directly equated, such as Odum and Rainaud (2003), similar results are found. This type of information would be good to make clear in the discussion.

Results:

Were there any significant order effects?

Figures 3 & 4: Would be good to label the outcomes directly in the figure rather than as A and B if possible.

Figure 2: Some measure of central tendency, like median, is also needed in addition to individual results. How were these individuals chosen?

Did the degree of discounting differ for food and money? I apologize if I have missed it, but I cannot find that statistical comparison in the results. This comparison seems like a critical piece of information to determine whether the basic results replicate many prior. Because different models fit the data best, a common metric like Area Under the Curve (AUC) could be a good way to do it.

Discussion:

The review paper by Odum et al. (2020) would be good to incorporate also into the introduction instead of just in the discussion as currently.

Small things:

Line 34: should be extent rather than extend

Line 36: remove comma
